# Regulatory Effects of Stubble Management on Leaf-Soil Carbon, Nitrogen, and Phosphorus Stoichiometric Relationships in *Caragana korshinskii*

**DOI:** 10.3390/plants15101584

**Published:** 2026-05-21

**Authors:** Wenli Ma, Min Yan, Hejun Zuo, Xue Chen

**Affiliations:** 1 Inner Mongolia Key Laboratory of Aeolian Physics and Desertification Control, Engineering College of Desert Control Science and Engineering, Inner Mongolia Agricultural University, 29 Erdos East Street, Hohhot 010018, China; 2 State Key Laboratory of Water Engineering Ecology and Environment in Arid Area, Inner Mongolia Agricultural University, 29 Erdos East Street, Hohhot 010018, China

**Keywords:** shrub restoration, stumping, ecological stoichiometry, rejuvenation, nutrient cycling

## Abstract

Restoration of degraded shrublands is a major challenge for combating desertification in arid and semi-arid regions. *Caragana korshinskii* Kom., a dominant sand-fixing shrub widely planted in northern China, often shows growth decline and structural degradation as stand age increases. Stubble management is widely used to rejuvenate degraded shrublands; however, its influence on nutrient cycling and carbon-nitrogen-phosphorus (C-N-P) stoichiometric coupling within the leaf-soil system remains unclear. Here, we conducted a two-factor field experiment in a 30-year-old degraded *C. korshinskii* plantation in the Kubuqi Desert, northern China, manipulating stubble height and stubble density. Moderate stubble height (10 cm) significantly increased leaf N concentration (27.37 g kg^−1^) and improved soil C and N availability, whereas higher stubble height (20 cm) led to elevated leaf N:P ratios (24.2), indicating stronger phosphorus limitation. In addition, all stubble density treatments significantly reduced leaf C:N, C:P, and N:P ratios. Among them, the two stubbled after one retained exhibited the most pronounced effect, with C:N and C:P decreasing to 14 and 273, respectively, and N:P to 20, suggesting an improved nutrient balance and allocation efficiency. Multivariate analyses showed that lower stubble heights combined with alternate-plant stubble patterns (H2D1 and H2D2) enhanced leaf-soil nutrient coupling and promoted coordinated recovery of C-N-P stoichiometry during regeneration. Overall, stubble management regulates shrub rejuvenation mainly by modifying leaf-soil nutrient coupling rather than single-element responses. It is recommended that, in the management of degraded *C. korshinskii* shrublands, a stubble height of approximately 10 cm combined with staggered cutting (alternate-plant or every two plants) be prioritized as an optimized management regime.

## 1. Introduction

Desert ecosystems constitute a fundamental component of terrestrial ecosystems, and their stability and the services they provide are closely linked to global ecological security and sustainable development [1]. Shrublands play a vital role in arid and semi-arid regions by offering windbreak and sand fixation, conserving soil and water, and preserving biodiversity, thereby forming an essential ecological barrier [2]. However, during long-term global desertification control efforts, both natural and planted shrublands have experienced growth decline and degradation. Shrubland degradation has become a widespread ecological problem in arid and semi-arid regions worldwide. This is characterized by plant senescence, reduced vegetation cover, decreased biomass, and simplified community structure, driven by multiple factors such as climate aridity, soil nutrient deficiency, water competition, pests, diseases, and human disturbance [3]. In this context, restoring shrubland ecosystems through appropriate management to enhance their ecological functions has become a central focus in combating desertification and promoting ecological restoration [4].

Within the uniquely fragile balance of desert ecosystems, *Caragana korshinskii* Kom. serves as a key pioneer species for sand fixation [5]. As a drought-tolerant, cold-resistant leguminous shrub with strong adaptability to poor soils and vigorous sprouting ability, *C. korshinskii* has been widely planted since the 1950s in desert and semi-desert regions of northern China for sand stabilization and ecological restoration [6]. With increasing stand age, however, planted *C. korshinskii* forests commonly exhibit growth stagnation, declining vitality, and structural degradation, manifested by branch dieback [7], slow new shoot regeneration, and reduced photosynthetic capacity and nutrient accumulation, often leading to large-scale mortality and a significant decline in sand-fixing and soil conservation functions [8]. To address the degradation of planted *C. korshinskii* shrublands, stubble management—a form of artificial intervention based on compensatory growth mechanisms that stimulates regrowth through the removal of aboveground biomass—has been widely applied for shrubland rejuvenation [9]. The effectiveness of stubble management is jointly influenced by multiple factors, including timing, intensity, frequency, and site conditions, with significant interactions among different management parameters. Appropriate combinations of stubble management practices can regulate stand structure, alleviate ecosystem degradation, and enhance primary productivity [10]. Previous studies have shown that stubble management promotes biomass accumulation and ecosystem function recovery by influencing sprouting capacity [11], plant height growth, and leaf functional traits [12,13,14]. Moderate stubble management can also regulate photosynthetic physiology and water-use processes, thereby improving water-use efficiency [15] and enhancing carbon sequestration potential [16]. At the soil level, well-designed stubble management can significantly increase soil microbial diversity and activity [17] and improve understory microhabitats, creating favorable conditions for vegetation restoration [18]. Despite its widespread application in restoring degraded *C. korshinskii* plantations, stubble management has often focused on single technical parameters—such as stubble height or strip treatment—due to engineering constraints and practical management considerations [19,20]. Systematic studies integrating multiple stubble management factors remain limited, particularly in long-term degraded shrublands in arid regions. Moreover, the nutrient allocation and coupling mechanisms within the leaf-soil system following stubble management have received insufficient attention, and the internal processes through which different stubble management patterns regulate ecosystem material cycling and stability remain unclear.

Carbon (C), nitrogen (N), and phosphorus (P) are essential elements for plant growth and development, and their concentrations and stoichiometric ratios directly affect plant physiological status and ecosystem functioning [21]. Ecological stoichiometry provides a quantitative framework for understanding nutrient limitation, energy flow, and element cycling by characterizing the distribution patterns of C, N, and P in organisms and their environment [22]. As the primary organ for photosynthesis and material exchange, leaf stoichiometric traits serve as key indicators of plant nutrient-use strategies and physiological adaptation [23]. The stoichiometric relationships between leaf and soil C, N, and P not only reflect plant nutritional status and soil nutrient supply capacity, but also indicate the efficiency and stability of nutrient cycling within ecosystems [24]. Recent studies have suggested that stubble management can alter internal resource allocation patterns. This process promotes the translocation and accumulation of photosynthates and nutrients such as N and P into newly formed leaves [25], implying that stubble management may improve plant function by regulating stoichiometric balance. However, whether integrated stubble management reorganizes the coupling mechanisms between plant and soil nutrient stoichiometry remains largely unexplored, particularly in long-term degraded shrubland ecosystems.

Therefore, this study focused on a 30-year-old degraded *C. korshinskii* plantation in the Kubuqi Desert and employed a two-factor controlled experiment to systematically evaluate the effects of stubble height and stubble density on C, N, and P concentrations and their stoichiometric characteristics in leaves and soils. Specifically, we aimed to address the following questions: (1) How do different stubble treatments alter leaf and soil C, N, and P concentrations and stoichiometric ratios? (2) Are the stoichiometric responses of leaves and soils to stubble management coupled? (3) Which stubble management pattern is optimal in terms of nutrient recovery and system stability? We hypothesized that (i) moderate stubble height would increase leaf N and soil N availability; (ii) alternate-plant stubble patterns would enhance leaf-soil C-N-P coupling; and (iii) combined height-density treatments would outperform single-factor interventions in restoring stoichiometric balance. By elucidating the stoichiometric mechanisms underlying stubble management regulation of the *C. korshinskii*-soil system, this study provides mechanistic insights for optimizing stubble practices and promoting the sustainable management of degraded shrublands in arid regions. This is the first study to integrate stubble height and density to evaluate leaf-soil C-N-P stoichiometric coupling in degraded *C. korshinskii* plantations.

## 2. Results and Analysis

### 2.1. Effects of Stubble Management on Leaf Ecological Stoichiometry of Caragana korshinskii

**Figure 1:**
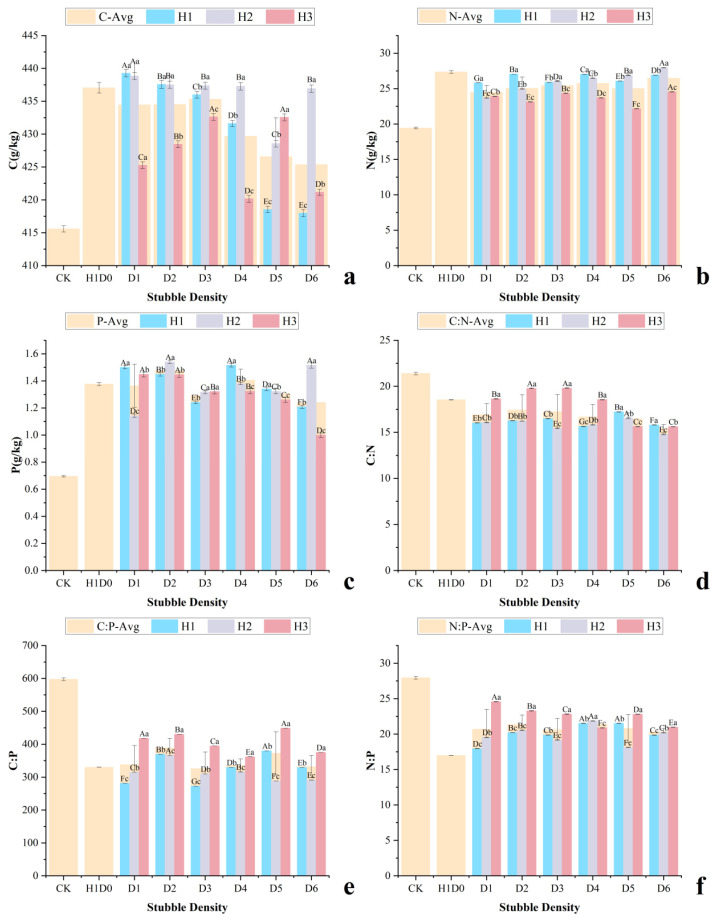
Leaf C, N, and P concentrations and stoichiometric ratios of *C. korshinskii* under different stubble management treatments. (**a**) Leaf C concentration; (**b**) Leaf N concentration; (**c**) Leaf P concentration; (**d**) Leaf C:N ratio; (**e**) Leaf C:P ratio; (**f**) Leaf N:P ratio. Values are means ± SD (*n* = 3). Uppercase and lowercase letters indicate significant differences among density and height treatments, respectively (*p* < 0.05).

Both stubble height and stubble density significantly affected leaf C, N, and P concentrations and their stoichiometric ratios (Figure 1). Leaf C concentrations remained relatively high across treatments and showed a stable-to-decreasing trend with increasing stubble height. No significant difference was observed between H1 and H2 (437.12 and 436.49 g kg^−1^, respectively; *p* > 0.05), whereas leaf C under H3 significantly declined to 426.37 g kg^−1^ (*p* < 0.01), representing a reduction of 2.5%. Leaf N and P concentrations exhibited unimodal responses to stubble height. Leaf N peaked under H2 (27.37 g kg^−1^), increasing by 10.4% and 14.8% compared with H1 and H3, respectively. Leaf P was highest under H1 (1.41 g kg^−1^), slightly decreased under H2, and declined significantly under H3 (1.27 g kg^−1^; *p* < 0.05). Consequently, leaf C:N and C:P ratios increased markedly under H3, reaching 18.4 and 345.7, respectively, which were 9.8% and 18.6% higher than those under H1. The leaf N:P ratio increased sharply to 24.2 under H3, exceeding the commonly accepted threshold for phosphorus limitation in terrestrial plants (N:P > 16).

From the perspective of density regulation, compared with the control (CK), all stubble density treatments significantly reduced leaf C:N, C:P, and N:P ratios. The D3 treatment showed the strongest effect, reducing C:N and C:P to 13.7 and 272.5, respectively, with an N:P ratio of 19.9. Substantial reductions were also observed under D6 and D4, where C:N decreased to approximately 14.4 (reductions of 32%) and C:P declined to 291.37 and 329.4, respectively, although these effects were slightly weaker than those under D3. Complete stubble (H1D0) significantly lowered all stoichiometric ratios but to a lesser extent than low-intensity density treatments. In contrast, D2 and D5 showed relatively moderate reductions in C:N and C:P, with N:P ratios remaining between 20.2 and 21.5.

### 2.2. Effects of Stubble Management on Soil Ecological Stoichiometry of Caragana korshinskii

**Figure 2:**
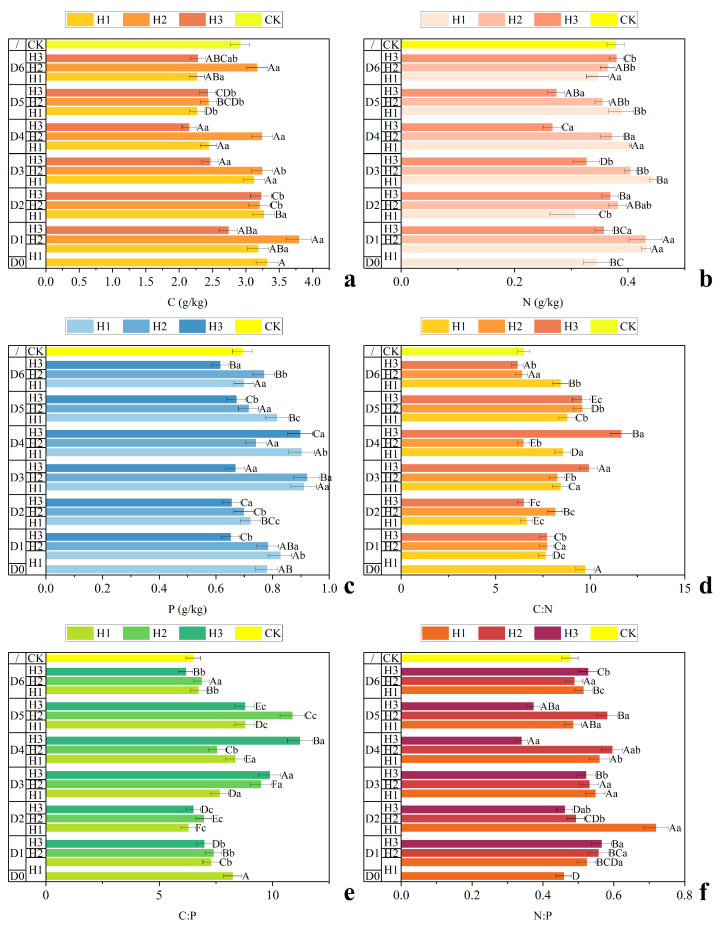
Soil C, N, and P concentrations and stoichiometric ratios of *C. korshinskii* plantations under different stubble management treatments. (**a**) Soil C concentration; (**b**) Soil N concentration; (**c**) Soil P concentration; (**d**) Soil C:N ratio; (**e**) Soil C:P ratio; (**f**) Soil N:P ratio. Values are means ± SD (*n* = 3). Uppercase and lowercase letters indicate significant differences among density and height treatments, respectively (*p* < 0.05).

As shown in Figure 2a–c, stubble height exerted significant effects on soil C, N, and P concentrations. From H1 to H2, soil C increased by 14.3% (from 3.12 to 3.56 g kg^−1^) and soil N increased by 11.7% to 0.40 g kg^−1^ (*p* < 0.05), whereas soil P increased only slightly (5.4%) and was not statistically significant (*p* > 0.05). When stubble height increased further to H3, soil C, N, and P concentrations all declined, with soil C showing the largest decrease (−21.8%). Figure 2d–f indicate differential responses of soil stoichiometric ratios to stubble height. Soil C:N reached its minimum at H2 (8.85), representing a 9.1% reduction relative to H1 (*p* < 0.05). In contrast, soil C:P and N:P peaked at H2, reaching 8.85 and 0.54, respectively, corresponding to increases of 7.4% and 17.6% compared with H1.

Overall, stubble management significantly altered soil nutrient balance in the *C. korshinskii* plantations. Compared with the control (CK), stubble treatments generally increased soil C:N and C:P ratios. The highest values of C:N (11.6) and C:P (11.2) were observed under the H2D6 treatment, whereas H1D4 maintained a relatively low C:N ratio (6.5). Soil N:P ratios across all treatments ranged from 0.34 to 0.72, which were far below the global average threshold for terrestrial plants (N:P = 16). The lowest N:P ratio occurred under H2D6 (0.40), while the highest value was observed under H1D4 (0.72).

### 2.3. Relationships Between Leaf and Soil C, N, and P Concentrations and Stoichiometric Ratios Under Different Stubble Management Treatments

Two-way ANOVA results (Table 1) demonstrated that stubble height exerted significant effects on leaf and soil C, N, and P concentrations and their stoichiometric ratios (*p* < 0.01). Except for soil C, stubble density significantly affected leaf C, N, and P concentrations and their stoichiometric ratios, as well as soil N and P concentrations and soil stoichiometry (*p* < 0.01). Significant interactive effects between stubble height and stubble density were observed for all leaf and soil C-N-P variables and their stoichiometric ratios (*p* < 0.01), indicating a strong coupling between management intensity and nutrient regulation.

**Figure 3:**
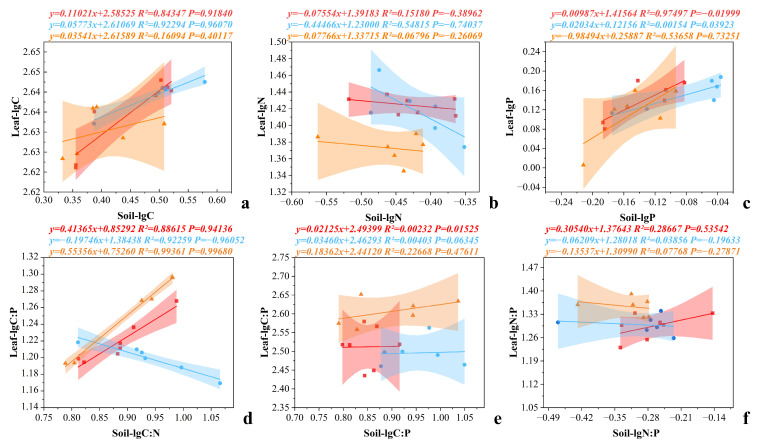
Standardized major axis (SMA) regression analysis of the relationships between leaf and soil C, N, and P concentrations and stoichiometric ratios under different stubble heights. (**a**) Relationship between leaf C and soil C concentrations; (**b**) relationship between leaf N and soil N concentrations; (**c**) relationship between leaf P and soil P concentrations; (**d**) relationship between leaf C:N and soil C:N ratios; (**e**) relationship between leaf C:P and soil C:P ratios; and (**f**) relationship between leaf N:P and soil N:P ratios. Each point represents a treatment combination. Red, blue, and yellow lines and equations represent the fitted relationships between leaf and soil C, N, and P concentrations under stubble heights of 0, 10, and 20 cm, respectively. The shaded areas in corresponding colors denote the 95% confidence intervals.

Regression analyses further revealed differential coupling patterns between leaf and soil nutrients under different stubble heights (Figure 3). Leaf C concentration showed no significant relationship with soil variables (*p* > 0.05), suggesting that leaf C stoichiometry was relatively insensitive to changes in soil nutrient status (Figure 3a). In contrast, leaf P concentration and C:P ratio exhibited significant differences among stubble height treatments (Figure 3c,e), although leaf P under H3 and leaf C:P under H2 and H3 showed no significant correlations with corresponding soil indicators (*p* > 0.05). Significant soil-leaf coupling was observed for leaf P under H1 and H2, and for leaf C:P under H1. Leaf N concentration was consistently and significantly correlated with soil N across all stubble height treatments. Moreover, leaf C:N under H2 and leaf N:P under H2 and H3 were significantly influenced by soil nutrient conditions, whereas leaf C:N under H1 and H3 and leaf C:P under H1 showed weaker or inconsistent responses. Overall, these results indicate that stubble management regulates leaf-soil nutrient coupling in a height-dependent manner, with moderate stubble heights enhancing soil-plant nutrient linkages and optimizing stoichiometric balance.

### 2.4. Relationships Between Leaf and Soil Nutrient Status and Stoichiometric Characteristics Under Different Stubble Management Treatments

**Figure 4:**
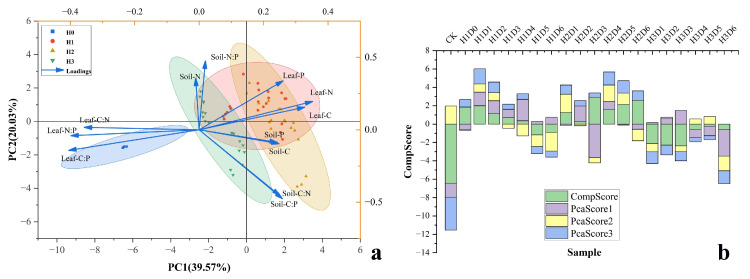
Effects of different stubble management treatments on leaf-soil C, N, and P stoichiometric characteristics of *C. korshinskii*. (**a**) Principal component analysis (PCA) ordination of stubble management treatments; The blue, red, yellow, and green ellipses represent the 95% confidence intervals for different stubble-height treatments (H0–H3) (**b**) Comprehensive PCA scores of leaf-soil C, N, and P stoichiometric characteristics under different treatments. Values represent weighted composite scores, with higher values indicating stronger rejuvenation effects following stubble management.

To clarify the integrated effects of stubble height and stubble density on leaf-soil stoichiometric characteristics, principal component analysis (PCA) was performed using 12 variables, including leaf and soil C, N, and P concentrations and their stoichiometric ratios. The PCA results indicate that stubble management significantly influenced the overall stoichiometric scores of leaf and soil systems (Figure 4a). Three principal components with eigenvalues greater than 1 were extracted. The first principal component (PC1) explained 39.57% of the total variance, the second (PC2) explained 20.03%, and the third (PC3) explained 13.05%, with a cumulative variance contribution of 72.65%, indicating that the PCA effectively captured the integrated leaf-soil nutrient status. PC1 was primarily associated with Leaf C:P, Leaf N:P, Leaf C:N, Leaf N, and Leaf C, accounting for the highest variance contribution (30.3%). Higher scores along PC1 reflected improved leaf nutrient status and overall shrub growth performance. In contrast, PC2 and PC3 were mainly related to soil nutrient variables, including Soil C:N, Soil C:P, Soil C, Soil P, Soil N:P, and Soil N, highlighting the contribution of soil nutrient availability to shrub nutrient regulation. Weighted PCA scores, calculated based on component loadings and variance contributions, revealed clear differences among stubble treatments. Treatments H1D1 and H2D4 exhibited significantly higher comprehensive stoichiometric scores, whereas the control (CK) and H3D3 showed relatively low scores. These results indicate that moderate stubble management can effectively improve leaf nutrient structure, enhance soil nutrient availability, and strengthen the stability and coupling of leaf-soil nutrient cycling in artificial *C. korshinskii* plantations of the Kubuqi Desert.

**Figure 5:**
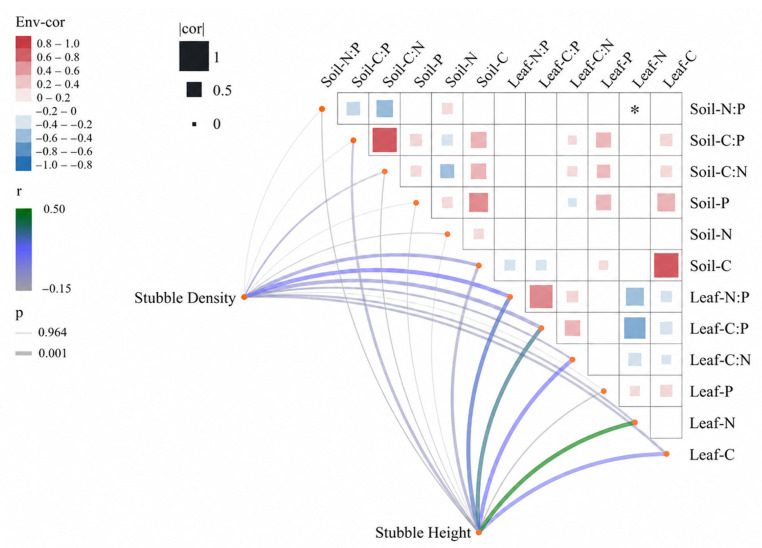
Relationships among leaf and soil C, N, and P concentrations, their stoichiometric ratios, and stubble management treatments. Blue and red color intensities indicate the strength of positive and negative correlations, respectively. Line color and thickness represent correlation significance based on Mantel’s *p*-values and Mantel’s r values. (*) indicate *p* < 0.05.

Leaf C, N, and P concentrations and their stoichiometric ratios were significantly correlated with soil C, N, and P concentrations and soil C:P ratios (Figure 5). In particular, leaf N concentration showed significant positive correlations with soil C:P and C:N (*p* < 0.05), suggesting that enhanced soil nutrient balance may promote plant C and N uptake, thereby regulating leaf C:P and N:P ratios. Leaf C concentration was positively correlated with soil N:P, C:N, and C:P, whereas soil C concentration was negatively correlated with leaf N:P, C:N, and C:P (*p* < 0.001), indicating that limited soil C availability may intensify nutrient constraints and induce adaptive adjustments in leaf stoichiometry. Stubble height was significantly associated with both leaf and soil nutrient indicators, highlighting its dominant role in regulating C-N-P dynamics in *C. korshinskii* plantations of the Kubuqi Desert. The significant interactions between stubble height and stubble density further demonstrate that changes in leaf and soil nutrient concentrations and stoichiometric characteristics are jointly driven by integrated stubble management strategies. These results provide strong evidence for coordinated plant-soil nutrient adaptation during shrub rejuvenation following stubble management.

## 3. Discussion

### 3.1. Effects of Different Stubble-Cutting Treatments on Leaf-Soil C, N, and P Stoichiometric Relationships and Ecological Adaptation Strategies of Caragana korshinskii

Ecological stoichiometric characteristics are key indicators reflecting plant growth strategies and nutrient limitation status. Under different degrees of rejuvenation management, plants adjust nutrient uptake and allocation among organs in response to external disturbances, thereby altering their ecological stoichiometric traits. The present study demonstrates that stubble-cutting disturbance significantly reshaped the nutrient cycling pathways within the leaf-soil system of *C. korshinskii* plantations in the Kubuqi Desert.

Stubble height emerged as a critical factor regulating leaf nutrient status, with our results strongly confirming the hypothesis that a moderate stubble height would increase leaf and soil nitrogen availability. Our results show that stubble-cutting had no significant effect on leaf C concentration, indicating that leaf carbon remains relatively stable under short-term disturbance due to its primary structural function in plant tissues [26]. In contrast, leaf N and P concentrations, which are important indicators of plant nutrient status, were significantly higher under low and moderate stubble heights than under high stubble height, suggesting improved nutrient accumulation under reduced cutting heights. This pattern may reflect increased nutrient demand during shoot regeneration following stubble-cutting disturbance. Higher N and P concentrations may be associated with the nutrient requirements of newly regenerated tissues [27]. Moreover, leaf C:N and C:P ratios, which reflect plant growth rate and nitrogen-use efficiency, were significantly higher under the high stubble height treatment (H3) compared with the low stubble height treatments (H1 and H2). This suggests that plant growth under H3 was constrained relative to low stubble treatments, indicating that lower stubble heights, particularly the moderate H2 intensity, can alleviate intrinsic growth limitations and promote higher leaf N accumulation in *C. korshinskii* leaves [28]. CK exhibited extremely severe phosphorus limitation, which was slightly alleviated under the H1D4 and H2D4 treatments but still persisted, indicating a pronounced phosphorus limitation on plant growth. This may be associated with the retention of older branches under high stubble height, which could reduce overall nutrient-use efficiency during regeneration. In contrast, under the strip-based spatial arrangement, compared with the alternate-plant stubble pattern, retaining a moderate level of old branches may reduce resource loss associated with senescent tissues. Meanwhile, the strip-based arrangement may improve resource availability and reduce intraspecific competition during regeneration.

Crucially, this validation of enhanced nitrogen availability extends from aboveground tissues to the belowground soil environment, as stubble-cutting density influenced nutrient stoichiometry by altering canopy closure and intraspecific competition [29]. Compared with the control (CK), all stubble-cutting treatments significantly reduced leaf C:N and C:P ratios. By removing part of the stand, stubble-cutting improved light availability and soil water accessibility, alleviating nutrient competition caused by excessive stand density. A decrease in leaf C:N implies relatively lower investment in structural carbon and increased synthesis of functional proteins, which may favor active plant growth [30]. Soil, as the primary nutrient source for plants, is highly sensitive to external disturbances. In alignment with our hypothesis regarding soil nutrient enrichment, moderate cutting significantly enhanced soil carbon and nitrogen resources while maintaining soil C:N at a relatively low level. Correlation analysis further confirmed a positive relationship between leaf N concentration and soil C:N, suggesting that stubble-cutting may alter soil nutrient availability and nutrient-use patterns, which in turn promotes N accumulation in aboveground tissues. As the growth substrate for plants and a reflection of ecosystem nutrient supply capacity, soil nutrient dynamics can be regulated by plant growth following stubble-cutting through changes in organic matter decomposition and nutrient transformation processes [31]. Consistent with previous studies [32], stubble-cutting may influence soil nutrient transformation processes. Changes in soil elemental composition following stubble-cutting may be associated with multiple ecological processes. Removal of aboveground biomass alters litter input, root turnover, and rhizosphere microbial activity, thereby affecting soil organic matter decomposition and nutrient cycling. In arid desert ecosystems, reduced canopy closure after moderate stubble-cutting can improve soil aeration and precipitation infiltration, which may stimulate microbial mineralization and enhance nitrogen availability. Meanwhile, lower plant density decreases intraspecific competition for soil nutrients and water, contributing to temporary nutrient accumulation in the rhizosphere. However, excessive cutting intensity may also increase soil exposure and nutrient loss through wind erosion and surface evaporation, particularly in sandy desert environments such as the Kubuqi Desert. Consequently, soil resources were optimized under moderate cutting but declined under excessive cutting or high stubble retention, providing definitive evidence that an intermediate cutting intensity maximizes soil nitrogen availability while avoiding risks associated with over-cutting. However, soil N:P ratios were generally extremely low (<1), far below the global average; this indicates that nitrogen (N) acts as the primary limiting nutrient, despite soil P being relatively enriched in the Kubuqi Desert. Nevertheless, low stubble height treatments (H1 and H2) enabled plants to utilize these limited soil nutrients more efficiently, thereby maintaining ecosystem stability in aging plantations.

### 3.2. Effects of Different Stubble-Cutting Treatments on Plantation Rejuvenation and Ecosystem Function Maintenance of Caragana korshinskii

Distinct differences were observed in the number and vigor of regenerative growth points under different stubble heights. Previous studies have suggested that lower stubble heights may increase the dependence of regenerating shoots on stored reserves because a larger proportion of photosynthetic tissues is removed during cutting [33]. The lower the stubble height, the greater the removal of photosynthetic tissues, and thus the higher the dependence on stored reserves and the associated nutrient consumption. Under the H1 treatment, all aboveground photosynthetic organs were removed, forcing regeneration to rely entirely on root reserves, which may temporarily increase physiological stress during early regeneration. This condition persists until newly formed shoots can conduct effective photosynthesis and transport assimilates back to the root system, during which plants may experience weakened growth or even mortality due to insufficient energy supply. Previous studies have reported that stubble-cutting may influence bud activation and regeneration patterns through changes in apical dominance and hormonal regulation [34]. This hormonal shift stimulates the emergence of numerous new shoots from the plant base or root collar. Stubble height directly determines the number and spatial distribution of dormant buds on residual stems, thereby regulating sprout abundance and vigor [35]. In the H1 treatment, all aboveground buds were removed, and regeneration relied solely on adventitious buds at the root collar or roots, which initiate slowly and are limited in number, leading to sluggish early regrowth. In contrast, the H2 treatment retained a greater number of dormant axillary buds on the stems, which were rapidly activated following the release of apical dominance and developed into new shoots. Although buds retained under the H3 treatment could sprout relatively quickly, their higher position and potential senescence often resulted in weaker shoot vigor, with new growth more likely to develop into lateral rather than dominant shoots. Stubble height also influenced plant water-use dynamics [36]. In the H1 treatment, wounds were located at the root collar; despite their small size, the complete loss of aboveground tissues may severely increase physiological stress during early regeneration. The H2 treatment, by retaining part of the stem, reduced overall wound area and transpiration loss, thereby maintaining better water balance and alleviating water stress during regeneration. In contrast, the long residual stumps under H3 continued to require additional maintenance costs, while new shoots emerging from higher positions experienced longer transport pathways, creating physiological disadvantages. Additionally, internal resource competition may arise between old stumps and new shoots.

As a dominant species for soil and water conservation and windbreak establishment in the Kubuqi Desert, *C. korshinskii* is commonly planted in belt-like arrangements [37]. Initial planting densities typically range from 1665 to 2500 individuals per hectare [38] or follow specific spacing configurations. However, with increasing stand age, unmanaged plantations often exhibit growth stagnation, increased branch mortality, and soil water depletion due to excessive density. Therefore, this study applied three stubble-cutting patterns—alternate-plant cutting, alternate-belt cutting, and clear-cutting—to regulate shrub growth.

Although clear-cutting is the most efficient rejuvenation method and facilitates subsequent management, it entails high ecological risks due to short-term vegetation removal, habitat destruction, and reduced stress resistance of regenerating seedlings. In contrast, alternate-plant cutting preserves ecosystem functions to a greater extent by selectively treating individual shrubs, maintaining seed sources, and sustaining complex microhabitat structures. The coexistence of retained and cut shrubs creates an uneven-aged structure closer to natural conditions, benefiting understory vegetation and fauna and enhancing ecosystem resilience and long-term stability. The D1 treatment (cutting every other plant) achieved a balanced half-stand renewal, with retained plants maintaining stand stability and supporting assimilating tissue production, while cut plants regenerated from the base. This promoted coordinated stand development and balanced resource allocation without excessive thinning or crowding.

**Figure 6:**
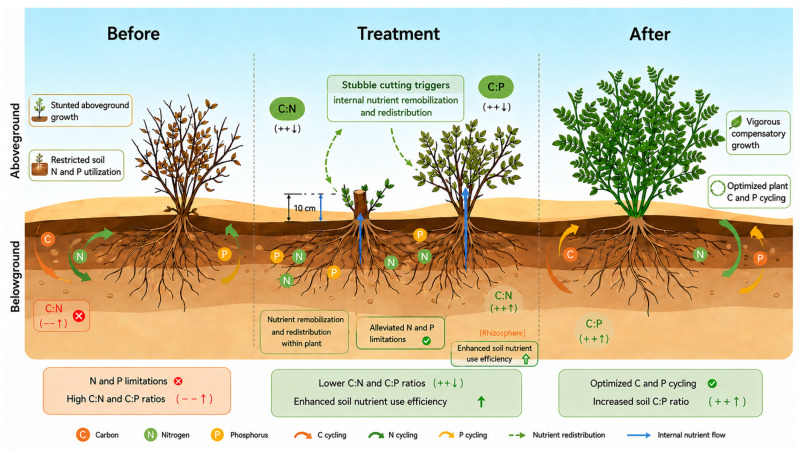
Mechanism of enhanced nutrient redistribution and utilization efficiency in *C. korshinskii* induced by 10 cm stubble height. “×” represents N and P limitations, “√” represents optimized C and P cycling, and “↑” represents enhanced soil nutrient use efficiency. Taking the 10 cm stubble height as an example, stubble cutting induces a three-stage process: initial growth limitation under low soil N and P availability, followed by nutrient remobilization that decreases plant C:N and C:P ratios (++↓) and enhances soil nutrient use efficiency, and finally compensatory growth characterized by strengthened C and P cycling and elevated soil C:P ratio (++↑).

These findings are generally consistent with our hypothesis that alternate-plant stubble patterns would enhance leaf-soil C-N-P coupling; by maintaining a functional vegetative buffer alongside regenerating individuals, this spatial arrangement optimizes reciprocal resource dynamics between the stand and the substrate. Furthermore, our findings generally support the third hypothesis that combined height-density treatments would outperform single-factor interventions in restoring stoichiometric balance. This is clearly demonstrated, as principal component analysis (PCA) and comprehensive scoring indicated that treatment combinations such as H1D1 and H2D4 exhibited significantly higher overall ecological benefits than other treatments, highlighting the synergistic effects of coupling cutting intensity with spatial density regulation. These findings suggest that management based on initial planting density strongly influences the ecological functioning of mature stands and that appropriate stubble-cutting strategies are essential. High stubble height not only restricted shrub rejuvenation but also failed to optimize leaf nutrient concentrations. In contrast, lower stubble heights substantially increased leaf and soil nutrient pools, effectively preserving basal dormant bud viability while eliminating senescent tissues to enable rapid vegetative regeneration. Given the characteristics of belt-shaped plantations, complete clear-cutting may cause short-term soil exposure and increase wind erosion risk [39]. Patchy or strip-cutting patterns such as “cutting every other plant” or “cutting one plant after two” maintained high nutrient-use efficiency in this study, further confirming the enhanced leaf-soil C-N-P coupling under alternate-plant management. These approaches may reduce excessive canopy closure and alleviate resource competition within the stand while retaining part of the stand as a windbreak, thereby preserving ecosystem stability. In conclusion, based on the coupled leaf-soil stoichiometric mechanisms identified in this study, a stubble-cutting regime combining a low stubble height (0–10 cm) with alternate-plant cutting patterns is recommended for achieving efficient nutrient recovery and long-term ecosystem stability in *C. korshinskii* plantations in the Kubuqi Desert (Figure 6). This strategy effectively resolves conflicts between stand aging and excessive density, optimizes leaf C:N:P stoichiometric balance, and may provide a suitable management strategy for sustainable plantation restoration. We therefore recommend promoting the “low stubble height + alternate-plant cutting” model in degraded shrubland restoration to replace conventional clear-cutting practices, thereby maintaining vegetation continuity, windbreak and sand fixation functions, and biodiversity. However, the underlying physiological and microbial mechanisms were not directly measured in this study and therefore require further investigation.

## 4. Materials and Methods

### 4.1. Study Area

The Kubuqi Desert, the seventh largest desert in China, extends in a long and narrow belt along the southern bank of the “Ordos Loop” of the Yellow River. It represents one of the most spatially extensive belt-shaped deserts in the country and is the only active desert located within China’s semi-arid region. The desert exhibits pronounced spatial gradients in natural environmental conditions. The study area is situated at the northern margin of the central Kubuqi Desert, on the northern Ordos Plateau in Inner Mongolia, China. Topography generally slopes from south to north and from west to east, with a mean elevation of approximately 1300 m a.s.l. The regional ecosystem transitions from desert in the south to arid steppe in the north, reflecting a clear ecological gradient. The area is characterized by abundant solar radiation, with a mean annual sunshine duration exceeding 3100 h. The mean annual air temperature is approximately 7 °C, and the average frost-free period is about 149 days. Long-term mean annual precipitation is 348.3 mm, with more than 70% occurring between July and September. In contrast, mean annual potential evaporation reaches 2506.3 mm, approximately 7.2 times the annual precipitation, indicating extremely severe water limitation. The mean annual wind speed ranges from 3.2 to 3.3 m s^−1^, with maximum wind speeds up to 22 m s^−1^, resulting in strong aeolian activity and pronounced wind erosion processes.

**Figure 7:**
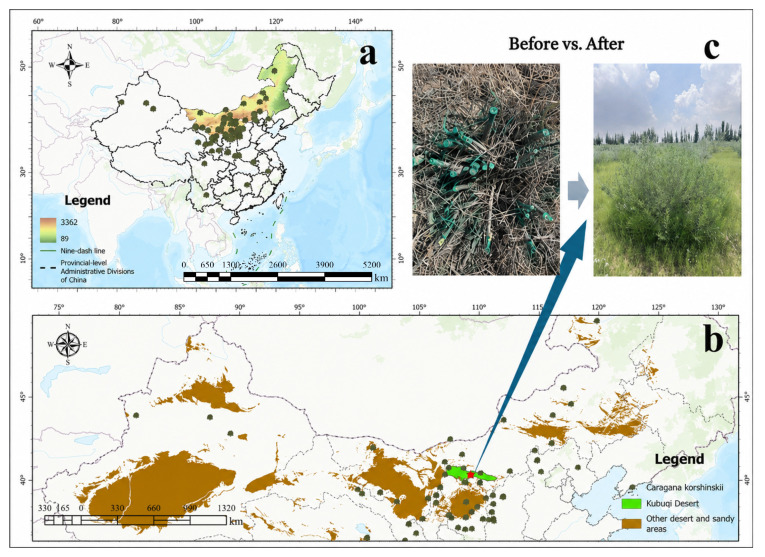
Overview of the study area. (**a**) Distribution of *C. korshinskii*; (**b**) Location of the study area; (**c**) Regrowth of *C. korshinskii* before (March) and after (August) stubble treatment. Administrative boundaries and geographic data were sourced from the Ministry of Natural Resources of China and CAS. Photographs in (**c**) were taken by the authors.

The study area was located in a typical *C. korshinskii* plantation (>30 years old) at the northern margin of the central Kubuqi Desert (Figure 7). Since 1977, this region has undergone extensive sand control and desertification mitigation, resulting in the establishment of various artificial vegetation communities that have contributed substantially to ecological restoration. However, parts of the *C. korshinskii* shrublands have experienced marked growth decline and structural degradation with increasing stand age, reaching moderate to severe degradation stages. The selected site is representative of degraded plantations in the region and is characterized by flat terrain and large, continuous patches, which facilitate the implementation of multiple stubble management treatments. Located within a fenced management area, the site experiences minimal external disturbance and is suitable for long-term comparative experiments and monitoring.

### 4.2. Experimental Design and Sampling

**Figure 8:**
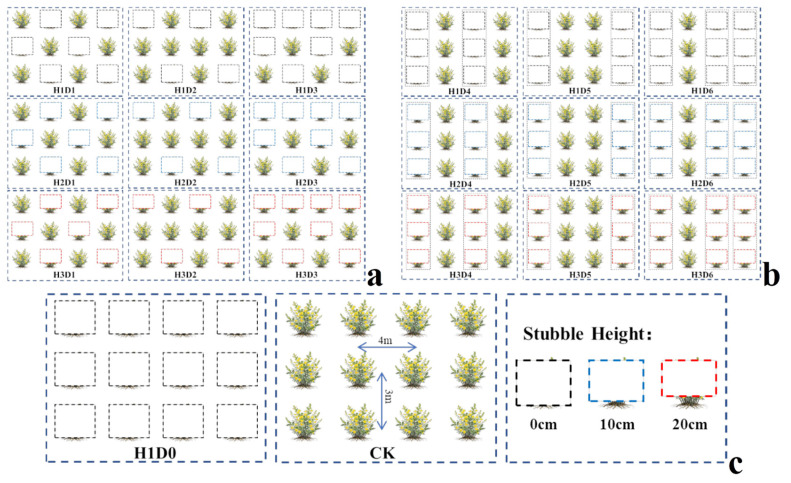
Schematic illustration of stubble management treatments. (**a**) Alternate-plant stubble pattern; (**b**) Alternate-row stubble pattern; (**c**) Complete stubble, control (CK), and stubble height treatments. The dashed boxes indicate varying stubble heights according to color: black for 0 cm, blue for 10 cm, and red for 20 cm.

The experiment was conducted in a typical degraded *Caragana korshinskii* plantation (30 years old) in the Kubuqi Desert, China (40.37° N, 109.46° E). The soil at the study site is classified as Kastanozems. To ensure consistent climatic characteristics and site conditions, all sampling plots were located within the same stand. All plots had similar site conditions, no previous management, and comparable growth status, with a shrub spacing of 3 m × 4 m. In this study, stubble density was regulated through spatial pattern manipulation, including individual-based (plant-level) and strip-based stubble treatments. Considering the interactive effects between stubble height and stubble density, stubble management treatments were applied in late March 2025 using a factorial design combining stubble height and stubble density. Stubble heights were set at 0 cm (H1), 10 cm (H2), and 20 cm (H3), together with six density patterns: alternate-plant stubble (D1), one stubbled after two retained (D2), two stubbled after one retained (D3; one plant cut for every two retained), alternate-row stubble (D4; alternate rows cut), one stubbled row after two retained (D5; one row cut after every two retained), and two stubbled rows after one retained (D6; two rows cut after every one retained). Complete stubble (H1D0) and an untreated control (CK) were also included. Each restoration mode included no fewer than 10 plants, and a total of 20 plots (30 m × 30 m each) were established with 5 m buffer zones between plots (Figure 8).

Leaf and soil sampling was conducted in late August 2025, representing the first growing-season response following stubble management implemented in late March 2025. By this time, the regrowth of stubbled plants had fully developed. To ensure the independence of experimental replicates, three spatially separated replicate sites were selected within the *C. korshinskii* plantation, with each site serving as a fully independent biological replicate. At each site, a 10 m × 10 m plot was established, and soil samples were collected using a five-point sampling method. Five shrubs subjected to the same treatment were selected for sampling. After removing surface litter and plant residues, soil samples were collected from five depth intervals (0–20, 20–40, 40–60, 60–80, and 80–100 cm). Soil samples from the same depth across the five shrubs were composited. Subsequently, the five depth layers were further mixed to obtain a depth-integrated sample per plot. Samples were air-dried in a ventilated room, ground, sieved (2 mm and then 0.15 mm), and stored at room temperature for subsequent analyses.

By the sampling period, the stubbled plants had fully regenerated their canopy. Within each plot, representative *C. korshinskii* individuals with consistent growth status were selected for leaf sampling. Ten healthy, fully expanded mature leaves were collected from the current-year shoots of at least three shrubs and pooled into one composite sample per plot. Leaf samples were collected in labeled bags and kept at approximately 4 °C during transport. In the laboratory, leaves were rinsed with deionized water and surface-dried with absorbent paper. Samples were heated at 105 °C for 30 min and then oven-dried at 60 °C to constant weight. The dried tissues were ground and passed through a 0.25 mm sieve prior to nutrient analysis.

### 4.3. Sample Analysis and Statistical Analysis

Laboratory analyses of plant and soil samples were conducted in October 2025. Organic carbon in leaf and soil samples was determined using the potassium dichromate oxidation method with external heating. Total nitrogen was measured using an automatic Kjeldahl analyzer (VAPODEST 200, C. Gerhardt GmbH & Co. KG, Königswinter, Germany) following H_2_SO_4_-H_2_O_2_ digestion, and total phosphorus was determined by the molybdenum blue colorimetric method. Leaf and soil C:N, C:P, and N:P ratios were calculated on a mass basis.

All statistical analyses were performed using IBM SPSS Statistics 27. One-way and two-way analyses of variance (ANOVA) were applied to evaluate the effects of different stubble management treatments on leaf and soil C, N, and P concentrations and their stoichiometric ratios. Duncan’s multiple range test was used for post hoc comparisons, with significance set at *p* < 0.05. Standardized major axis (SMA) regression analysis and principal component analysis (PCA) were performed using Origin 2024 to examine scaling relationships and major patterns of variation in leaf-soil stoichiometric characteristics among treatments. Mantel tests were conducted in R 4.5.1 to assess the relationships between leaf and soil C-N-P variables and their responses to different stubble treatments. Figures and spatial distribution maps were prepared using ArcGIS Pro 3.5.4.

## 5. Conclusions

(1) Regulatory Mechanism of Stubble Height on Resource Availability and Stoichiometry

Stubble height serves as a primary control variable driving the nutrient reorganization of the *C. korshinskii* leaf–soil system, with 10 cm (H2) identified as the optimal threshold for plantation rejuvenation. At this height, leaf N concentration reached its maximum (27.37 g kg^−1^), while soil C and N concentrations also increased relative to the other treatments, indicating improved nutrient availability and utilization under moderate stubble height. In contrast, the exacerbation of phosphorus limitation under high stubble (H3, N:P > 24.2) demonstrates that improper retention heights constrain nutrient recovery. These findings indicate that moderate stubble height optimizes soil nutrient supply, effectively optimizes soil nutrient supply and alleviates nutrient constraints.

(2) Spatial stubble-cutting patterns further influenced nutrient allocation and stoichiometric balance

Selective individual-plant cutting outperformed strip-cutting in optimizing nutrient allocation and reducing leaf stoichiometric ratios. Specifically, under the D3 treatment (cutting one plant for every two retained), leaf C:N and C:P decreased to 14 and 273, respectively. Individual-plant cutting patterns (D1 and D2) significantly reduced leaf C:N and C:P by up to 32%. This suggests that individual-level management is more effective than strip-cutting in improving nutrient cycling, thereby promoting a more balanced stoichiometric relationship within the plantation.

(3) Restoration of the *C. korshinskii* Leaf–Soil Nutrient Relationship Driven by the Interaction of Stubble Height and Cutting Density

The restoration of the *C. korshinskii* leaf–soil nutrient relationship resulted from the interactive effects of stubble height and cutting density rather than from a single factor. Stubble height and cutting density jointly restructured leaf-soil nutrient relationships. Leaf N concentration was positively correlated with soil C:N and C:P, and tighter coupling between leaf stoichiometry and soil nutrients was observed under H1 and H2 treatments. Two-way ANOVA further indicated that the interaction between stubble height and cutting density exerted highly significant effects on leaf and soil C, N, and P contents as well as their stoichiometric ratios (*p* < 0.01), and was more influential than either factor alone.

(4) A New Management Framework for Long-Term Maintenance of Degraded Plantations

Overall, the integrated management model of “low stubble height (0–10 cm) + selective individual-plant cutting” effectively improved nutrient allocation and stoichiometric balance in degraded plantations. By optimizing the C:N:P stoichiometric balance, this strategy achieves a harmony between nutrient turnover efficiency and regenerative potential. These findings provide a quantifiable technical pathway for the precision restoration of shrublands in the Kubuqi Desert and similar arid regions worldwide, contributing a new management framework for the long-term maintenance of artificial plantations.

## Figures and Tables

**Table 1:** Two-way ANOVA results for the effects of stubble height and stubble density on leaf-soil C, N, and P concentrations and stoichiometric characteristics of *C. korshinskii*.

Anzeige	Stubble Height			Stubble Density			Height × Density		
	*df*	*F*	*η* ^2^	*df*	*F*	*η* ^2^	*df*	*F*	*η* ^2^
C	2	17.195 **	0.475	6	0.68	0.097	12	7.856 **	0.713
N		9.495 **	0.333		18.498 **	0.745		12.469 **	0.797
P		85.996 **	0.819		23.159 **	0.785		29.585 **	0.903
C:N		6149.721 **	0.997		2952.729 **	0.998		6466.544 **	1
C:P		9948.069 **	0.998		1933.216 **	0.997		4472.651 **	0.999
N:P		8.849 **	0.318		15.945 **	0.716		8.970 **	0.739

C: R^2^ = 0.994; N: R^2^ = 1.000; P: R^2^ = 0.988; C:N: R^2^ = 1.000; C:P: R^2^ = 1.000; N:P: R^2^ = 1.000; ** *p* < 0.01.

## Data Availability

The original data presented in the study are openly available in [Mendeley Data] at [10.17632/6tr5rp4c9t.1].
